# Seed and Color Preferences of Wild Carrion Crows From Cafeteria Experiments

**DOI:** 10.1002/ece3.70944

**Published:** 2025-02-05

**Authors:** Amal Chantoufi, Amanda Marques Canário, Tilwenn Baud, Clément Vallé, Alice Baux, Frédéric Jiguet

**Affiliations:** ^1^ Agroscope Plant‐Production Systems Nyon Switzerland; ^2^ Centre d'Ecologie et des Sciences de la Conservation (CESCO), Muséum National d'Histoire Naturelle, CNRS Sorbonne Université Paris France

**Keywords:** bird damage, carrion crows, color coatings, preference, sunflower

## Abstract

Birds can cause significant damage to agricultural fields, notably in Europe, where corvid species like carrion crows ( *Corvus corone* ) and rooks ( *Corvus frugilegus* ) target spring crops, posing challenges for conservation and wildlife management. Among targeted crops, sunflower and maize suffer the highest levels of damage from corvids' foraging. While both lethal and non‐lethal strategies are employed to mitigate bird damage, their effectiveness is limited and context‐dependent. Consequently, there is growing interest in identifying natural bird repellents. To improve our understanding of corvid food selection and explore potential non‐lethal management strategies, we conducted (1) cafeteria trials with five common seed types (maize, sunflower, soy, pea, and wheat) and (2) two‐choice color tests with maize seeds in four colors (blue, green, orange, and red) on an urban population of carrion crows accustomed to human presence. Results indicated a marked preference for sunflower and wheat, while soy and pea were largely avoided, and maize was moderately consumed. The crows also demonstrated a preference for green‐colored maize over blue, orange, red, and natural maize seeds. These findings suggest that strategic planting of preferred crops like wheat alongside sunflower or maize may help mitigate damage. Further, incorporating colors into repellent seed coatings could reinforce the aversive learning process in birds, although color may play a less significant role in food selection when seeds are sown.

## Graphical abstract

In the context of significant damage caused by corvids to spring crops, there is a need for sustainable management strategies informed by a better understanding of their ecology. Our study addresses this gap by focusing on the seed and color preferences of wild carrion crows.

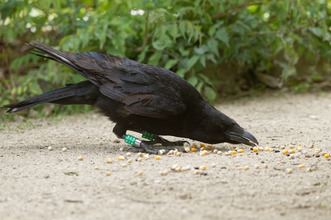

As humans and wildlife frequently share the same ecological niche, they inevitably vie for resources such as space and food (Araneda, Ohrens, and Ibarra 2022). Urbanization and agricultural expansion are bringing humans closer to natural and protected areas, leading to increased interactions (Araneda, Ohrens, and Ibarra 2022; Htay et al. 2022). This proximity, coupled with natural habitat disturbances, has heightened human–wildlife conflicts. Such conflicts emerge when animals consume or damage resources utilized by humans, resulting in significant economic losses (Canavelli et al. 2014; Furlan et al. 2021). In agricultural ecosystems, crops initially intended for human or livestock consumption can become crucial food resources for wildlife (Htay et al. 2022). Thus, generalist and abundant species foraging in these habitats and causing damage to crops are often classified as pests (Jiguet 2020b; Klug et al. 2023).

Birds can cause substantial economic losses through their foraging activities (Canavelli et al. 2014; Furlan et al. 2021) in various types of agricultural fields such as field crops, paddy fields, and horticultural crops. These losses result from direct consumption or partial damage that lead to spoilage (Klug et al. 2023). Omnivorous and granivorous birds such as doves, parrots, parakeets, sparrows, crows, and blackbirds are often reported to be the origin of damage to field crops (Linz et al. 2011; Sausse and Lévy 2021). In Europe, corvid damage to spring crops represents a significant challenge for farmers (Furlan et al. 2021; Sausse and Lévy 2021). Sunflower and maize are the most targeted crops (Furlan et al. 2021; Destrez et al. 2022). For instance, 90% of bird damage reported in 2021 in France affected sunflower plantations (Martin‐Monjaret and Sausse 2021). Birds damage the seeds and early stages of these crops (Esther, Tilcher, and Jacob 2013; Furlan et al. 2021), imposing substantial economic losses.

In response to these challenges, various bird damage control methods have been implemented to mitigate agricultural losses (Klug et al. 2023), usually divided into lethal and non‐lethal strategies. The use of lethal methods involves strategies such as shooting, nest destruction, poisoning, and trapping (Betz Heinemann et al. 2020; Linz et al. 2011) to reduce bird numbers. These approaches are rarely monitored and display short‐term effectiveness when applied to abundant populations with high fecundity and dispersion capacity (Betz Heinemann et al. 2020; Sausse et al. 2021). Additionally, they raise ethical and environmental concerns, and their cost‐effectiveness was only recently questioned (Jiguet 2020b).

Ongoing research is exploring alternative, non‐lethal strategies for bird management to balance crop protection and wildlife conservation (Day et al. 2012; Sausse et al. 2021; Destrez et al. 2022). Traditional approaches, such as auditory deterrents (e.g., propane cannons and distress calls) and visual scare tactics (e.g., balloons and scarecrows) (Linz et al. 2011), are commonly employed but exhibit only short‐term effectiveness due to rapid habituation by birds (Esther, Tilcher, and Jacob 2013; Klug et al. 2023). Non‐lethal methods also include sowing practices' adjustments, such as deeper seed placement and increased spacing between seeds (Canavelli et al. 2014; Huang et al. 2023). Repellents represent another widely used category of non‐lethal deterrents. These include chemical deterrents (Esther, Tilcher, and Jacob 2013) and aversive conditioning techniques (Werner, Kimball, and Provenza 2008), applied through seed spraying or coating. Natural plant‐derived substances with low toxicity and reduced ecological impact have also been investigated as potential repellents (Hile et al. 2004; Avery et al. 2005; Linz et al. 2007; Klug et al. 2023). However, these substances have mainly been tested on captive populations or wild corvids held in captivity (Hile et al. 2004; Avery et al. 2005; Linz et al. 2007; Day et al. 2012; Esther, Tilcher, and Jacob 2013; Destrez et al. 2022). These studies often overlook critical factors such as birds' ability to choose food under natural conditions and individual feeding preferences, both of which are likely essential for evaluating the effectiveness of damage prevention measures (Linz et al. 2011; Day et al. 2012). Furthermore, the responses of free‐ranging populations can differ significantly from those of captive birds due to confounding factors absent in controlled environments (Day et al. 2012; Esther, Tilcher, and Jacob 2013; Sausse and Lévy 2021).

Birds have complex ecology and behavior that need to be taken into account when implementing damage prevention methods (Guarino 1972; Jiguet 2020b), especially concerning food selection (Sausse et al. 2021). Corvids are mid‐to large‐sized birds that have great behavioral and diet plasticity (Benmazouz et al. 2021). They are common in urban areas and, given their ability to exploit abundant anthropogenic food sources (Matsyura, Zimaroyeva, and Jankowski 2016; Benmazouz et al. 2021), they are responsible for an important part of crop damage in France (Martin‐Monjaret and Sausse 2021). As a result, they are strongly regulated, with an estimated 380,000 carrion crows ( *Corvus corone* ) culled annually (Aubry et al. 2016).

To gain further understanding of corvids' food selection in crop fields, we organized experiments on seed and color preferences, using the opportunity to study an urban population of wild carrion crows used to being fed by humans or foraging near them. The objectives of the experiments were (1) to determine the seed types that wild crows prefer and (2) to test if they display a particular aversion for a given color. Concretely, we conducted (1) cafeteria trials with five common seed species (maize, sunflower, soy, pea, and wheat) and (2) two‐choice preference tests using corn seeds in four different colors besides its natural color. Overall, the results could help identify potential attractive seeds that could be used as “trap” crops and aversive colors to mitigate corvid damage to crops.

## 1 Study Area

The study was conducted in Jardin des Plantes, Paris, France (48.84° N, 2.36° E), a large public park managed by the National Museum of Natural History. This urban park hosts a relatively large population of carrion crows that are familiar with the constant human presence and easily approachable, and are studied by ringing since 2015 (Lequitte‐Charransol, Robert, and Jiguet 2024).

## 2 Methods

### 2.1 Experimental Design

#### 2.1.1 Seed Preference Tests

Carrion crows' seed preferences were assessed using multiple‐choice preference tests, commonly referred to as cafeteria trials. A total of 35 trials were conducted. Five seed species were presented simultaneously to crows: soy ( *Glycine max* ), sunflower ( *Helianthus annuus* ), pea ( *Pisum sativum* ), wheat ( *Triticum aestivum* ), and maize ( *Zea mays* ). Sunflower and maize were chosen because they are highly damaged by corvids at sowing (Furlan et al. 2021; Sausse and Lévy 2021) while sown soy and wheat seeds may also be subject to damage by feeding corvids (Govorushko 2014; Kennedy and Connery 2008). Finally, pea was chosen because of its use as a “trap crop” in a crop protection project called “Peacor” where a pea strip is sown in corn or sunflower plots to limit bird damage (Limagrain Europe n.d.).

**FIGURE 1:**
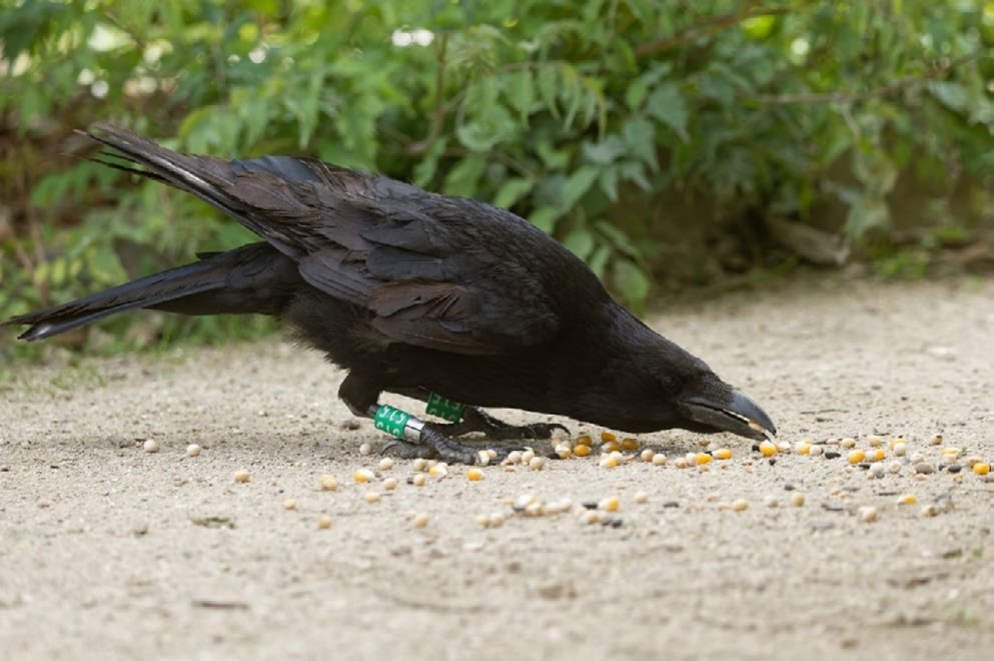
A ringed carrion crow (G515) feeding on seeds in a multiple‐offer trial at Jardin des Plantes, Paris (author of photographs: M.L. Pamart).

Birds were presented with 40 of each of the five seed species at the same time mixed together within a 30 × 30 cm area and were continuously observed throughout the experiment (Figure 1). For each test, we selected a different site than the previous one, where at least three individuals were already present. This process was repeated two to three times daily from the 6th to the 22nd of March 2023. The number of each type of seed eaten and the number of individuals present during each experiment categorized as < 5, between 5 and 10, or > 10 individuals were recorded.

An experiment was considered to be valid when 40 to 160 seeds of the 200 seeds (corresponding to 20% and 80% of the 200 seeds available) were consumed. This range was chosen to ensure a sufficient level of consumption for preference analysis and avoiding excessive depletion of the seed offer. The tests did not have a standardized duration because of the variability in bird group size. Out of the 35 multiple‐offer seed preference trials carried out, 27 were considered valid. Six experiments were discarded due to the consumption of seeds by pigeons, and 2 were excluded because the number of seeds did not meet the validity threshold.

#### 2.1.2 Color Preference Tests

**FIGURE 2:**
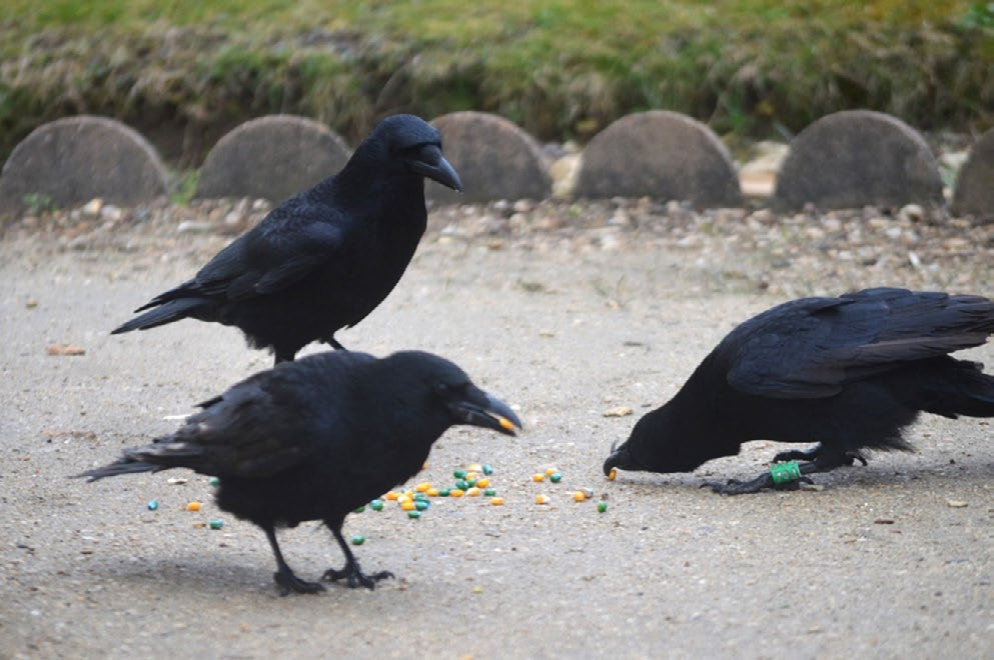
Carrion crows feeding on maize in a two‐choice blue color trial at Jardin des Plantes, Paris (author of photographs: T. Baud).

We studied color preference in free‐ranging carrion crows using two‐choice tests. Maize was chosen as a test substrate because it ranks as the second most targeted crop by bird attacks, can be easily coated with food dyes, and is consumed by urban crows. Food dyes (E*xxx*) were used to coat maize seeds. Colors used were green (5.7 mL of Quinoline Yellow E104 and Brilliant blue E133 per 100 g), red (2 mL of Carminic acid E120 per 100 g), orange (mix of 1.4 mL of Yellow 5 (Tartrazine) E102 and 0.95 mL of Cochineal Red E124), and blue (3.8 mL of E133 per 100 g). Birds were offered 40 colored (treatment) and 40 uncolored seeds (control) simultaneously (Figure 2). Treatments were presented in a random order, ensuring that the same color was never tested consecutively, and each test was conducted at a different location from the previous one within the Jardin des Plantes, where at least three individuals were already present. As in seed preference tests, no standardized duration was fixed, and tests were considered to be valid when at least three crows participated. Tests were concluded by deterring the birds once a visual estimate indicated that approximately 20 to 60 seeds from the total presented had been consumed. The mean duration of color tests was 5.18 ± 2.61 min.

Data were collected from the 6th of March to the 7th of April 2023, and a total of 71 color tests were carried out, including 20 tests for green and 17 tests each for blue, orange, and red.

### 2.2 Statistical Analyses

All statistical analyses were performed in R, version 4.3.3 (R Core Team 2024) and the significance level considered was 0.05.

#### 2.2.1 Seed Preference Tests

Results of the multiple‐offer trials were analyzed using a negative binomial mixed‐effects model (NBMM) due to over‐dispersed counts using the R package *lme4* (Bates et al. 2015). The number of each seed species eaten was the response variable. Explanatory fixed effects were seed species and Julian date and their interaction. A date effect was included to account for increased willingness to forage over time. The seed species with the highest frequency of consumption was used as the reference treatment. To account for the within‐test dependence (i.e., dependence between seed counts relating to the same multiple‐offer preference test), a test ID random effect was included. Differences in seed choice were determined using pairwise comparisons of marginal means with a Tukey correction.

#### 2.2.2 Color Preference Tests

We modeled the proportion of colored seeds eaten as a function of seed color and Julian date and their interaction using a generalized linear model (GLM) with a binomial error structure and a logit link (McCullagh and Nelder 1989) from the R base package. Bonferroni pairwise comparisons (“emmeans” function in *emmeans* package) (Lenth et al. 2018) were used to adjust *p* values during multiple comparisons.

In both seed and color tests, the number of participating individuals was not taken into account, and preferences were evaluated at the population level. We did not control for pseudo‐replication (due to potential multiple observations on the same birds) because we considered that the study population consisted of at least 100 individuals. Including unringed individuals and occasional visitors, the total number of birds frequenting the site likely reaches one hundred daily. To comfort this estimate, approximately 250 first‐calendar‐year birds are ringed annually at Jardin des Plantes. They all visit the site at least once, though they are not all present at the same time; more details on local apparent survival and movement rates can be found in Lequitte‐Charransol, Robert, and Jiguet (2024).

For both models, we checked the distribution of the residuals and the homogeneity of variances (Faraway 2006). The backward elimination procedure was then used to sequentially simplify the model for interactions that were not significant. The importance of the eliminated variable was determined using likelihood ratio tests.

## 3 Results

### 3.1 Seed Preference Tests

**FIGURE 3:**
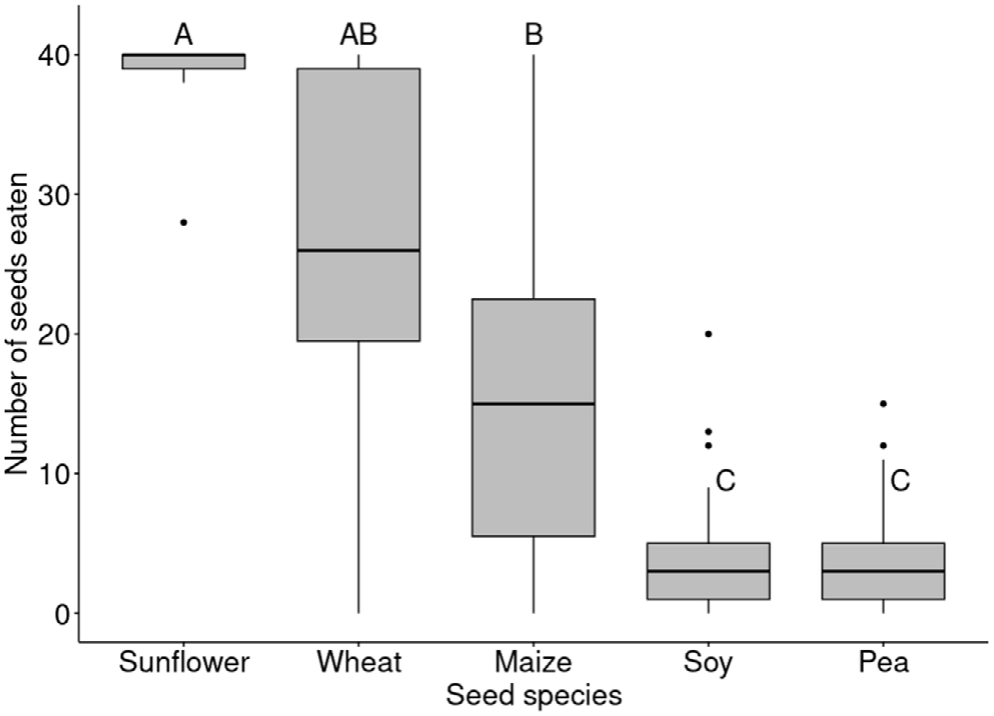
Boxplot indicating the number of seeds consumed by carrion crows in multiple‐offer seed preference trials, where 40 of each of the five seed species were offered together. Letters represent significant post hoc comparisons (NBMM with sequential Tukey correction). Boxplots that do not share a letter have significantly different means.

The results of the study are presented as the mean number of seeds of the 40 seeds available consumed by the participating individuals for each seed species ± SD. The exact number of individuals was not recorded; most tests (18) fell into the 5–10 individuals' category. Sunflower seeds were the most preferred seed (39.2 ± 2.32 SD) followed by wheat (26 ± 13.6 SD) and maize (15.7 ± 12.4 SD) while peas (3.85 ± 3.98 SD) and soy (4.11 ± 4.73 SD) were the least consumed seed species. The interaction between seed species and time was not significant (*χ* ^2^4 = 6.55, *p* = 0.16). Additionally, consumption of seeds did not significantly change throughout time (*χ* ^2^_1_ = 0.36, *p* = 0.54; Table S1). Multiple comparison probabilities adjusted using the Tukey method showed that sunflower was preferred over maize, soy, and pea (Figure 3). Maize was preferred over soy and pea (Figure 3).

### 3.2 Color Preference Tests

On average, 6.8 ± 1.68 SD carrion crows were present during each experiment and consumed seeds. We found no significant interaction between color and Julian date (*χ* ^2^_3_ = 5.37, *p* = 0.146; see Table S2 for details).

**FIGURE 4:**
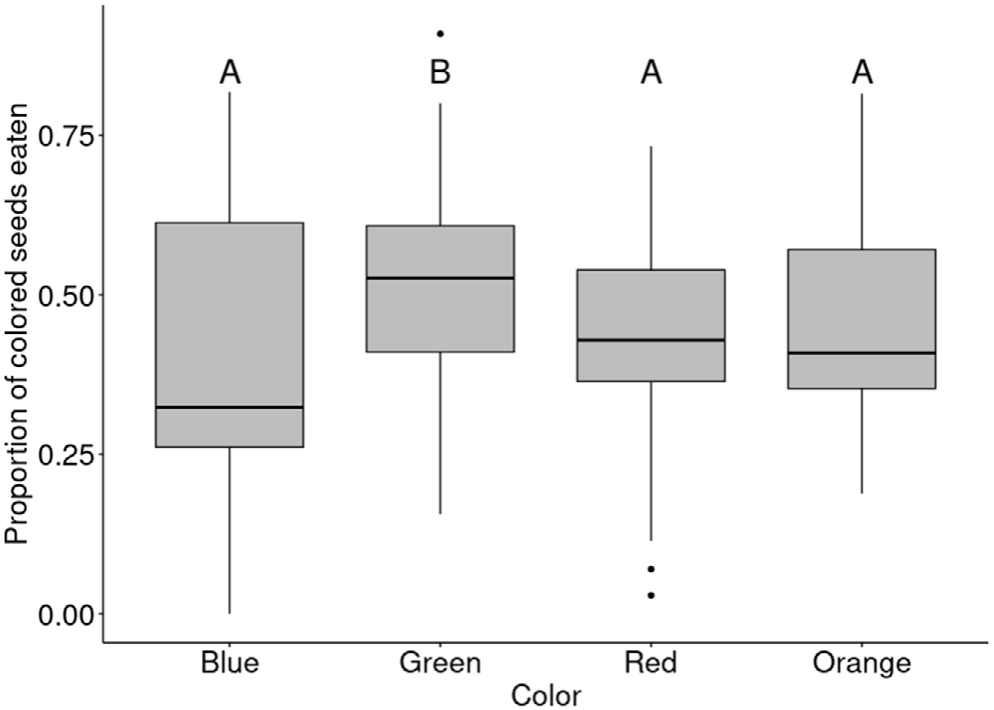
Boxplots of the proportion of colored maize seeds consumed by carrion crows in two‐choice color preference trials. Letters represent significant post hoc comparisons (GLM for binomial data with sequential Bonferroni correction). Boxplots that are not sharing a letter have significantly different means.

We were interested in knowing how the proportion of colored seeds eaten differed according to the color (*χ* ^2^_3_ = 33.62, *p* < 0.001). Crows showed a significant preference for green‐colored maize over blue, orange, and red (Figure 4). Other color comparisons were not significant after the sequential Bonferroni correction. Additionally, carrion crows had a significantly higher probability of selecting green‐colored maize over natural maize (53.2% ± 1.92% SE) in comparison with orange (44.8% ± 2.24% SE, *p* < 0.05), red (39.8% ± 1.97% SE, *p* < 0.001), and blue (38.6% ± 2.12% SE, *p* < 0.001). In contrast, no significant preference for orange, red, or blue maize over natural seeds was observed (*p* > 0.05).

**FIGURE 5:**
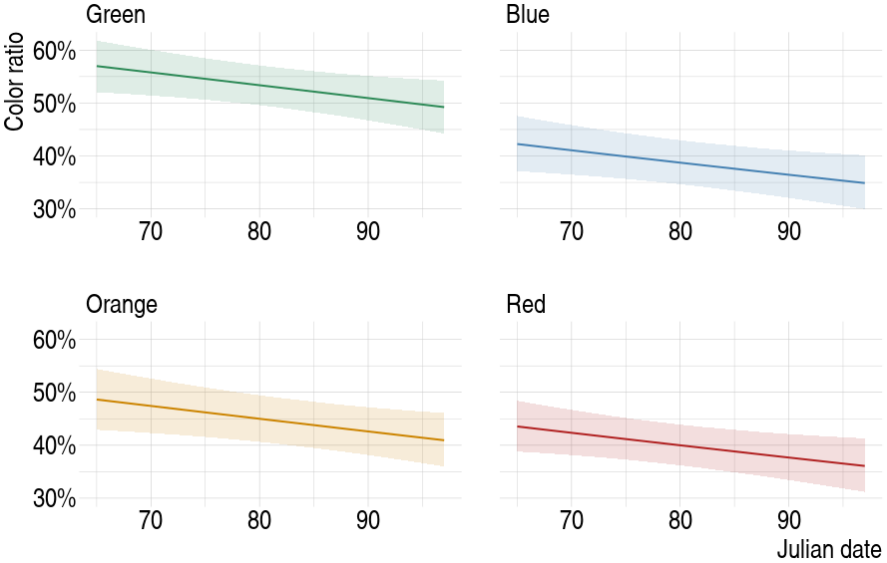
Relationship between the proportion of colored seeds consumed and the Julian date per color tested, as predicted by the binomial GLM with 95% confidence intervals.

Consumption of colored seeds relative to natural seeds significantly decreased throughout time for all colors tested (*χ* ^2^_1_ = 5.43, *p* < 0.05, Figure 5).

## 4 Discussion

In this study, we provide insights on crow seed and color preferences in an urban context.

### 4.1 Seed Preferences

Sunflower and wheat appear to be preferred seed species, while soy and pea seem to be avoided. Maize is a secondarily preferred food item. Carrion crows had similar seed preferences in rural habitats for sunflowers as it is the main crop damaged by birds in France (Sausse and Lévy 2021) and Western Switzerland (Résultats de l'enquête sur les dégâts d'oiseaux, Agroscope 2021, unpublished data). According to Sausse and Lévy (2021), only *Corvidae* can consume the sown seeds. Tracking data (Movebank Study ID 1266784970) further suggest that urban carrion crows frequently visit rural areas, supporting the assumption that they are familiar with the seed species being offered. In contrast, wheat seems to be less consumed by crows, potentially because it is sown at a different time than sunflower, making the two crops unavailable simultaneously. Additionally, the higher sowing density of wheat compared to sunflower may further reduce the intensity of damage.

A high preference for sunflower and wheat seeds when presented with other food items is likely attributable to their common use as key ingredients in bird food mixtures sold commercially and frequently offered to crows by park visitors, while soy is rarely included (Lin 2005; Orros and Fellowes 2015). Additionally, maize and peas are frequently crushed when formulating a seed mix, making them less recognizable in whole form (Lin 2005). In Jardin des Plantes, where carrion crows are regularly fed by visitors year‐round, preference trials revealed that crows consumed seeds following a neophobic tendency (i.e., an initial distaste for unfamiliar food items), favoring familiar seed types over unfamiliar ones (Greggor et al. 2016). Furthermore, the “Carré Lamarck” section in the park, showcasing living collections of so‐called useful plants, including cereals (wheat, barley, millet, and triticale) and oilseed crops (flax, poppy, and sunflower) (Juhé‐Beaulaton 2022) reinforces this familiarity.

Selection might also be influenced by seed morphology in relation to mechanical digestion (Diaz 1994) or handling time (Schluter 1982). Sunflower and wheat seeds may be preferred simply because they can be easily removed from the feeding area and taken aside for consumption. Indeed, many birds prefer to grab a food item and transport it to a sheltered location for eating (Tvardíková and Fuchs 2012), a behavior we also observed during seed preference trials. In contrast, larger seeds (i.e., maize, peas and soy) require more time to flake and consume and may therefore be less favored.

Relocating birds by sowing attractive strips or spreading seeds figures among territorial management strategies to reduce pressure on sensitive crops (Sausse and Lévy 2021). In Europe, a diversion approach called “PEACOR” is tested to reduce bird damage on maize and sunflower fields (Limagrain Europe n.d.). A pea strip is sown adjacent to the crop to attract crows and pigeons *Columba* sp., given that peas are rich in proteins (INRA CIRAD AFZ 2017) and tend to be more appealing to birds during the breeding season. This approach may work for pigeon attacks (Robin, Ballanger, and Robert 2011) or when peas are at the seedling stage but matches our findings regarding crow seed preferences less. Future trials testing the efficacy of sowing maize and sunflower in wheat strips could be valuable to determine whether this approach helps to “dilute” the damage.

### 4.2 Color Preferences

Birds are also known to rely on visual cues when it comes to food choice (Werner, Kimball, and Provenza 2008; Destrez et al. 2022). As orange and non‐colored maize are visually similar, we anticipated them to be consumed to the same extent. However, the fact that red, orange, and blue colors are aposematic signals to birds (Pegram and Rutowski 2014) could explain the reduced interest in these colors compared to green seeds in our work. Moreover, we predicted blue seeds to be further rejected, given that this color was avoided by corvids in a study by Destrez et al. (2022). Finally, green is very common in nature and is probably not associated with negative cues, which could explain its preference by carrion crows.

Although corvids are highly neophobic (Miller et al. 2015; Greggor et al. 2016), the avoidance of a new color rapidly decreases (Destrez et al. 2022). In this study, carrion crow's aversion did not diminish, instead it increased for all tested colors. As Destrez et al. (2022) hypothesized, urban crows are likely to encounter differently colored objects and food originated from human activities. In Jardin des Plantes, birds often consume anthropogenic resources, thus the colors used might not have been entirely novel. Furthermore, several studies have already proven that fruit color preference has a great interindividual variation (Willson and Comet 1993). We did, indeed, notice that dominant individuals ate more seeds than subordinate ones, as we noted down ring numbers of those that participated in nearly all the tests. This could mean that our results mostly reflect the dominant's preferences, whereas, in fields, seeds are available across a large area allowing subordinate individuals to feed. Finally, the increased aversion detected when considering time could be explained by the presence of individuals who never tasted colored seeds. In fact, there is a large crow population and a high individual turnover within the park (Jiguet 2020a). Nevertheless, the existence of neophobia (Miller et al. 2015; Greggor et al. 2016) indicates that consumption of colored seeds will likely increase once every individual is familiar with the color. Conducting preference tests with ringed individuals would allow us to examine how the consumption of colored seeds changes with increased exposure to each color.

## 5 Management Implications

While the coloration of seeds is usually presented as a damage prevention strategy with ephemeral effectiveness (Destrez et al. 2022), incorporating colors into seed coatings may still enhance corvid aversion. Although color plays a lesser role in food selection once seeds are sown, it is still important to account for its potential influence when testing the efficacy of commercial repellent seed coatings, particularly those with distinct coloration, such as Korit 420 FS in orange (BAT Agrar n.d.) or Ibisio in purple (Bayer SeedGrowth n.d.).

Aversion to colors varies greatly between individuals, making the efficacy of these methods context‐dependent (Willson and Comet 1993). Numerous studies have highlighted the role of color cues in the conditioning process, underscoring the importance of visual stimuli in facilitating aversive learning in avian species (Lett 1980; Mason and Reidinger 1983; Werner, Kimball, and Provenza 2008). This suggests that experiments combining color cues and repellent flavors could offer valuable insights into the development and implementation of natural repellents for field crops.

Finally, it is essential to consider that this work was conducted in an urban context and not directly in fields. The confounding factors changing the birds' motivation may be different between urban and rural regions. For instance, at Jardin des Plantes, the number of tourists and the presence of alternative food sources likely influenced crows' seed and color preferences. Additionally, it would be interesting to confirm these findings by testing aversion to the same colors on other populations in different locations. Even so, our results contributed to the identification of potential attractive seeds and aversive colors to mitigate corvid damage to crops in Europe.

## Author Contributions

**Amal Chantoufi:** conceptualization (lead), data curation (equal), formal analysis (equal), investigation (supporting), methodology (equal), visualization (equal), writing – original draft (lead), writing – review and editing (equal). **Amanda Marques Canário:** conceptualization (supporting), data curation (equal), formal analysis (supporting), investigation (equal), methodology (supporting), writing – original draft (supporting). **Tilwenn Baud:** data curation (equal), formal analysis (supporting), investigation (equal). **Clément Vallé:** formal analysis (equal), methodology (equal), visualization (equal). **Alice Baux:** conceptualization (supporting), funding acquisition (lead), supervision (supporting), writing – review and editing (equal). **Frédéric Jiguet:** conceptualization (supporting), investigation (supporting), supervision (lead), writing – review and editing (equal).

## Ethics Statement

Seeds were colored with edible food dyes verified as harmless to birds; hence, the cafeteria experiments did not require referral to an ethics committee (the Cuvier ethics committee at the MNHN was indeed consulted and responded accordingly).

## Conflicts of Interest

The authors declare no conflicts of interest.

## Supporting information


Table S1.
Table S2.

## Data Availability

The data that support the findings of this study are available in Dryad at reference URL: http://datadryad.org/stash/share/Xr1baFtbPE3jHDRxGPWbGf_OzjnmuOD1qrvry‐CyK0s.
