# Supplementary material for: Seed and Color Preferences of Wild Carrion Crows From Cafeteria Experiments
Source: Ecol Evol. 2025 Feb 5;15(2):e70944. doi: 10.1002/ece3.70944 (PMC11799056; doi:10.1002/ece3.70944)
Supplement: Supplementary file 1 — Table S1. Table S2. [file ECE3-15-e70944-s001.docx]

**Supporting information on study results**

Table S1. Effect of seed species and Julian date on the number of consumed seeds. A NBMM is presented here with test ID included as a random factor. Significant fixed effects (P > 0.05) are bolded. Sunflower is the reference category.

|  | **Counts of seeds eaten** | | | | |
| --- | --- | --- | --- | --- | --- |
| *Predictors* | *Incidence Rate Ratios* | *std. Error* | *CI* | *Statistic* | *p* |
| type [Sunflower](Intercept) | 39.22 | 5.39 | 29.96 – 51.33 | 26.72 | **<0.001** |
| type [Wheat] | 0.66 | 0.13 | 0.45 – 0.96 | -2.17 | **0.030** |
| type [Maize] | 0.40 | 0.08 | 0.27 – 0.58 | -4.68 | **<0.001** |
| type [Pea] | 0.10 | 0.02 | 0.06 – 0.15 | -10.74 | **<0.001** |
| type [Soy] | 0.10 | 0.02 | 0.07 – 0.16 | -10.43 | **<0.001** |
| Scaled julian date | 1.05 | 0.08 | 0.90 – 1.21 | 0.60 | 0.548 |
| **Random Effects** | | | | | |
| σ^2^ | 0.42 | | | | |
| τ_00_ _ID_ | 0.02 | | | | |
| ICC | 0.04 | | | | |
| N _ID_ | 27 | | | | |
| Observations | 135 | | | | |
| Marginal R^2^ / Conditional R^2^ | 0.682 / 0.695 | | | | |

Table S2. Effect of color and julian date on total consumption. A GLM with binomial error structure is presented here. Green is the reference category.

| *Predictors* | *Odds Ratios* | *std. Error* | *CI* | *Statistic* | *p* |
| --- | --- | --- | --- | --- | --- |
| Color [Green] (Intercept) | 2.50 | 0.87 | 1.27 – 4.94 | 2.63 | **0.008** |
| Color [Blue] | 0.55 | 0.07 | 0.44 – 0.70 | -5.02 | **<0.001** |
| Color [Orange] | 0.71 | 0.08 | 0.57 – 0.90 | -2.83 | **0.005** |
| Color [Red] | 0.58 | 0.07 | 0.47 – 0.73 | -4.80 | **<0.001** |
| Julian Date | 0.99 | 0.00 | 0.98 – 1.00 | -2.33 | **0.020** |
| Observations | 71 | | | | |
